# Oral Health Knowledge, Attitudes, and Behaviors (KAB) of German Dental Students: Descriptive Cross-Sectional Study

**DOI:** 10.3389/fmed.2022.852660

**Published:** 2022-03-11

**Authors:** Abanoub Riad, Mayte Buchbender, Hans-Peter Howaldt, Miloslav Klugar, Martin Krsek, Sameh Attia

**Affiliations:** ^1^Department of Public Health, Faculty of Medicine, Masaryk University, Brno, Czechia; ^2^Department of Oral and Maxillofacial Surgery, University of Erlangen-Nuremberg, Erlangen, Germany; ^3^Department of Oral and Maxillofacial Surgery, Faculty of Medicine, Justus-Liebig-University, Giessen, Germany; ^4^Czech National Centre for Evidence-Based Healthcare and Knowledge Translation (Cochrane Czech Republic, Czech EBHC: JBI Centre of Excellence, Masaryk University GRADE Centre), Faculty of Medicine, Institute of Biostatistics and Analyses, Masaryk University, Brno, Czechia

**Keywords:** dental education, dental students, Germany, Hiroshima University – Dental Behavioral Inventory (HU-DBI), knowledge, attitudes, practices, oral health

## Abstract

Germany's 2030–oral health agenda incorporates behavioral targets such as twice-daily toothbrushing and routine dental check-ups. Given the professional and social roles of dentists in oral health promotion, the oral health-related knowledge, attitudes, and behaviors (KAB) of dentists and dental students became worth investigation. The present study was designed as a descriptive cross-sectional study that aimed to evaluate oral health KAB of German dental students using the Hiroshima University – Dental Behavioral Inventory (HU-DBI). A total of 508 dental students filled in the questionnaire, out of which 74.2% were females, 38.8% were clinical students, 11.4% reported tobacco smoking at least once week, 26.6% reported drinking alcohol at least once a week, and 82.9% reported suffering from problematic internet use. The overall HU-DBI score was high (7.67 ± 1.32), and it was slightly higher among females (7.70 ± 1.33) than males (7.59 ± 1.29), and gender-diverse students (7.33 ± 1.37). Clinical students (7.88 ± 1.26) had a significantly higher HU-DBI score, especially in the domain of oral health behaviors, compared with preclinical students (7.53 ± 1.34). A significant improvement in oral health behaviors and HU-DBI score was found between the third- *vs*. the fourth year, which corresponds to the period when prophylaxis, hygiene, and periodontology courses are delivered. Tobacco smoking was significantly associated with poor oral health knowledge, behaviors, and overall HU-DBI score. Problematic internet use and alcohol drinking had slightly lower HU-DBI scores. The findings of the present study call for early implementation of preventive dentistry elements in German curricula and addressing oral health needs of gender minorities in Germany by future epidemiologic studies.

## Introduction

In 2021, the German Dental Association (BZÄK) released the oral health goals of the health system in Germany for the year 2030, based on the best available epidemiologic evidence (1). The 2030–agenda incorporates both disease-related and health promoting-related objectives, e.g., a caries-free level of 90% for 3-year-olds and 12-year-olds, severe periodontal disease prevalence below 10% for middle-aged adults (35–44 years old), and improvement of oral health-related behaviors (1). The behavioral targets include (i) increasing the proportion of twice-daily brushing to be 87.5% among children, 85.3% adults, and 89.1% seniors, and (ii) increasing the proportion of the people who seek regular dental check-ups annually to be 86.9% among children, 75% adults, and 94.6% seniors (1, 2). The importance of behavioral interventions for oral health is underlined by the fact that oral diseases are multi-factorial in nature; and they are greatly influenced by several behaviors such as oral hygiene, oral healthcare seeking, tobacco smoking, and stress-coping (3, 4).

Dentists have a fundamental role in primary prevention through their social capacity for inducing and enhancing positive behavioral changes of their patients, families, and communities, as they are widely perceived as role models of oral hygiene (5–7). The public notion of dental professionals' superiority in terms of oral health is proven by the prevailing evidence that confirmed that oral health reported behaviors and clinical outcomes were significantly better among dentists than laypersons (8). In addition to oral hygiene, several health topics can be effectively addressed by dentists, including smoking cessation (9–13), physical activity (14–17), healthy nutrition (18–21), child neglect (22, 23), immunization (24–26), and hygiene (27–29). Like dentists, dental students tend to exhibit better oral health knowledge and attitudes than other university students, including healthcare students, e.g., medical, pharmacy, and nursing (30–33). Therefore, a long-standing hypothesis was laid down claiming that undergraduate curricula of dental schools can improve oral health attitudes and behaviors as collateral for equipping the students with the basic clinical skills and theoretical knowledge required to perform their future job (34–36).

Dental education in Germany is funded by the state except for one school (Witten-Herdecke) out of the 30 dental schools that are scattered all over the country (37). Dentistry programs last for 5 years and 6 months, as the last 6 months (the 11^th^ semester) are reserved for the state exam, which is compulsory to obtain the qualification degree. Like other European universities, the early semesters of German dental programs are predominantly occupied with basic medical science subjects; therefore, the first five semesters (from 1^st^ to 5^th^) are considered as “preclinical,” while the following five semesters (from 6^th^ to 10^th^) are considered as “clinical” because they accommodate the clinical-oriented subjects (37, 38).

The Hiroshima University – Dental Behavioral Inventory (HU-DBI) of Kawamura is a validated instrument for evaluating oral health-related knowledge, attitudes and behaviors (KAB) with reportedly high psychometric properties (35, 36). HU-DBI had been used by hundreds of studies that were conducted within the last 30 years and used the instrument either with (adoption) or without (adaptation) modifications (39–42). HU-DBI-based studies had revealed a lot about the oral health KAB of future dental professionals, i.e., dental students, in multiple regions and countries. Clinical students are supposed to have better oral health-related knowledge than their preclinical colleagues. Consequently, the oral health attitudes and behaviors of clinical students may get significantly improved (43–45). Female dental students tended to exhibit better oral health KAB than males using HU-DBI (46, 47).

The overarching aim of this study was to evaluate oral health-related KAB and its determinants among dental students in Germany. The primary objective was to estimate the oral health KAB of German dental students using HU-DBI. The secondary objectives were: (i) to explore the associations between oral health KAB and sociodemographic determinants such as gender and academic year, (ii) to investigate the role of dental curricula on oral health KAB, and (iii) to explore the associations between oral health KAB and risk behaviors, e.g., tobacco smoking and problematic internet use.

## Materials and Methods

### Design

The present study had been designed as a descriptive cross-sectional study utilizing a self-administered questionnaire (SAQ) that was developed and disseminated digitally through KoboToolBox (Harvard Humanitarian Initiative, Cambridge, MA, USA, 2021) (48). The study was carried out during the winter semester of the academic year 2021/2022, specifically between October 13^th^ and December 16^th^, 2021. The STrengthening the Reporting of OBservational studies in Epidemiology (STROBE) guidelines for cross-sectional studies had guided the execution and reporting of this study (49).

### Participants

The target population of the present study was dental students who were enrolled at German universities during the academic year 2021/2022. The study used a non-random sampling technique through pragmatic recruitment of the target population in two principal universities, which were Friedrich-Alexander-Universität Erlangen-Nürnberg (Nuremberg, Bavaria, Germany) and Justus-Liebig-Universität Gießen (Giessen, Hesse, Germany), in addition to approaching other German universities students through social media networks. The study was promoted during lectures and practical classes of all academic years in the two principal universities.

The students were able to access the SAQ through quick response (QR) codes and uniform resource locator (URL). The students were assured that their identity was anonymous and the decision to participate was completely voluntary which had no effect on their academic grading. The students who did not provide their consent to participate and those who had incomplete responses were excluded from the final analysis.

The required sample size was calculated using Epi-Info ^TM^ version 7.2.5 (CDC. Atlanta, GA, USA, 2021), and it was 413 students (50). The following assumptions were used: 5% error margin, 95% confidence level (CI), 50% outcome probability, 10% postulated invalid responses rate, and a target population size of 15,575 dental students in Germany based on the latest report of the Federal Statistical Office of Germany (Wiesbaden, Hesse, Germany) (51).

### Instrument

The German version of HU-DBI used in this study was produced by Wieslander et al. (52). The Medical Outcomes Trust (MOT) guidelines governed the process of translation, validation, and cross-cultural adaptation of the German HU-DBI version which exhibited satisfactory psychometric properties (53). Beside the original 20 dichotomous (agree/disagree) items of HU-DBI, the sociodemographic characteristics including gender, academic year, and university were included. Additional four dichotomous (agree/disagree) items were added inquiring about tobacco smoking “I consume tobacco at least once a week,” alcohol drinking “I drink alcohol at least once a week,” problematic internet use “I find myself using my smartphone/compute longer than I planned,” and regular dental check-up “I go to the dentist/ hygienist for regular check-up at least once a year” (54) (Table 1).

**Table 1:** Modified version of the Hiroshima University – Dental Behavioral Inventory (HU-DBI).

**No**.	**Question**	**Agree**	**Disagree**
1	I do not worry much about visiting the dentist.	□	□
**2**	**My gum tends to bleed when I brush my teeth**.	**□**	**□**
3	I worry about the color of my teeth.	□	□
**4**	**I have noticed some white sticky deposits on my teeth**.	**□**	**□**
5	I use a child sized toothbrush.	□	□
**6**	**I think that I cannot help having false teeth when I am old**.	**□**	**□**
7	I am bothered by the color of my gum.	□	□
**8**	**I think my teeth are getting worse despite my daily brushing**.	**□**	**□**
**9**	**I brush each of my teeth carefully**.	**□**	**□**
**10**	**I have never been taught professionally how to brush**.	**□**	**□**
**11**	**I think I can clean my teeth well without using toothpaste**.	**□**	**□**
**12**	**I often check my teeth in a mirror after brushing**.	**□**	**□**
13	I worry about having bad breath.	□	□
**14**	**It is impossible to prevent gum disease with tooth brushing alone**.	**□**	**□**
**15**	**I put off going to dentist until I have a toothache**.	**□**	**□**
**16**	**I have used a dye to see how clean my teeth are**.	**□**	**□**
17	I use a toothbrush which has hard bristles.	□	□
18	I do not feel I have brushed well unless I brush with hard strokes.	□	□
**19**	**I feel I sometimes take too much time to brush my teeth**.	**□**	**□**
20	I have had my dentist tell me that I brush very well.	□	□
21	I find myself using my smartphone/compute longer than I planned.	□	□
22	I consume tobacco at least once a week.	□	□
23	I drink alcohol at least once a week.	□	□
24	I go to the dentist/ hygienist for regular check-up at least once a year.	□	□

*The questions No. 1 – 20 are the original HU-DBI items and the questions in bold font are used to compute the overall score*.

Out of the 20 dichotomous items of HU-DBI, only 12 items are used to calculate the overall HU-DBI score, while the rest are considered as dummy items (55, 56). For each “agree” answer of the items no. 4, 9, 11, 12, 16, and 19 and “disagree” answer for the items no. 2, 6, 8, 10, 14, and 15, one point is added (36). The HU-DBI score ranges between 0 and 12, and the high score represents an improved overall oral health KAB. The knowledge-index score (K) is calculated by summing up items 2, 8, 10, 15, and 19. The attitudes-index score (A) is calculated by summing up items 6, 11, and 14. The behaviors-index score (P) is calculated by summing up items 4, 9, 12, and 16 (36, 55).

### Ethics

The study protocol was reviewed and approved by the Ethics Committee of the Faculty of Medicine, Masaryk University under the reference number 48/2019. The participating students had to provide their informed consent digitally before filling in the questionnaire. The present study was designed and conducted following the declaration of Helsinki for research involving human subjects (57). In addition, the general data protection regulation (GDPR) of the European Union (EU) guided the data storage and management process (58). No financial rewards or other incentives were involved in this study, and no identifying personal data was collected from the participants. The study participants were able to withdraw from the study at any point before submitting their responses to the digital SAQ.

### Analyses

The Statistical Package for the Social Sciences (SPSS) version 28.0 (SPSS Inc. Chicago, IL, USA, 2021) was used to perform all statistical tests (59). Firstly, Shapiro-Wilk test was used to verify whether the numerical variables were normally distributed or not with a significance level of ≤ 0.05. The overall score of HU-DBI was 12 points: 5 points for knowledge, 3 points for attitudes, and 4 points for behaviors. The descriptive statistics were performed to summarize the dataset; categorical and ordinal were described by frequencies (*n*) and percentages (%), and numerical variables were described by means and standard deviations (μ ± *SD*). The inferential statistics were used to test the proposed associations between the independent variables (sociodemographic and behavioral) and the dependent variables (oral health KAB). Chi-squared test (χ^2^), Mann-Whitney test (*U*), Kruskal Wallis (*H*), Jonckheere-Terpstra test (*JT*), and logistic regression analysis were used with confidence level (*CI*) of 95% and a significance level (*p-value*) of ≤ 0.05.

## Results

### Sociodemographic Characteristics

A total of 508 students had been included in the present study, of which females were the majority (74.2%), followed by males (24.6%), and gender-diverse (1.2%). The sample was nearly balanced over the five academic years with 311 (61.2%) preclinical students enrolled in 1st – 3rd year, and 197 (38.8%) clinical students enrolled in 4^th^ – 5^th^ year. The most contributing university was Friedrich-Alexander-Universität Erlangen-Nürnberg (49%), followed by Justus-Liebig-Universität Gießen (33.7%), Martin-Luther-Universität Halle-Wittenberg (6.3%), Eberhard Karls Universität Tübingen (2.4%), Universität Heidelberg (2.4%), and Universität des Saarlandes (2.4%). No missing data or empty responses were received (Table 2).

**Table 2:** Socio-demographic characteristics of German dental students responding to HU-DBI, winter 2021, (*n* = 508).

**Variable**	**Outcome**	**Frequency (*n*)**	**Percentage (%)**
**Gender**	Female	377	74.2 %
	Male	125	24.6 %
	Gender-diverse	6	1.2 %
**Academic year**	First Year	85	16.7 %
	Second year	114	22.4 %
	Third year	112	22 %
	Fourth year	112	22 %
	Fifth year	85	16.7 %
**Clinical experience**	Preclinical	311	61.2 %
	Clinical	197	38.8 %
**University**	Friedrich-Alexander-Universität Erlangen-Nürnberg	249	49 %
	Justus-Liebig-Universität Gießen	171	33.7 %
	Martin-Luther-Universität Halle-Wittenberg	32	6.3 %
	Eberhard Karls Universität Tübingen	12	2.4 %
	Universität Heidelberg	12	2.4 %
	Universität des Saarlandes	12	2.4 %
	Medizinische Hochschule Hannover	9	1.8 %
	Universität Leipzig	2	0.4 %
	Johannes Gutenberg-Universität Mainz	2	0.4 %
	Julius-Maximilians-Universität Würzburg	1	0.2 %
	Ludwig-Maximilians-Universität München	1	0.2 %
	Albert-Ludwigs-Universität Freiburg	1	0.2 %
	Technische Universität Dresden	1	0.2 %
	Universität Hamburg	1	0.2 %
	Christian-Albrechts-Universität zu Kiel	1	0.2 %
	Rheinisch-Westfälische Technische Hochschule Aachen	1	0.2 %

### General Health Behaviors

On asking the participants about their general health behaviors, 11.4% reported tobacco smoking at least once a week, 26.6% reported drinking alcohol at least once a week, and 82.9% reported suffering from problematic internet use. The male students (20%) reported to be significantly more engaged with smoking behavior (*p* = 0.004) than their female (8.8%) and gender-diverse peers (0%). Similarly, males (35.2%) reported to be significantly more engaged with alcohol drinking (*p* = 0.018) than female (24.1%) and gender-diverse students (0%). Contrarily, female (84.1%) and gender-diverse students (83.3%) had slightly higher levels of problematic internet use than their male peers (79.2%), which was not statistically significant (*p* = 0.365). The preclinical and clinical students did not report having significantly different tobacco smoking, alcohol drinking, or problematic internet use levels (Table 3).

**Table 3:** General health behaviors of german dental students responding to HU-DBI, winter 2021, (*n* = 508).

**Variable**	**Outcome**	**Female** **(*n* = 377)**	**Male** **(*n* = 125)**	**Gender-diverse** **(*n* = 6)**	***p-value***	**Preclinical** **(*n* = 311)**	**Clinical** **(*n* = 197)**	***p-value***	**Total** **(*n* = 508)**
Tobacco smoking	Yes	33 (8.8%)	25 (20%)	0 (0%)	0.004^*^	37 (11.9%)	21 (10.7%)	0.669	58 (11.4%)
	No	344 (91.2%)	100 (80%)	6 (100%)		274 (88.1%)	176 (89.3%)		450 (88.6%)
Alcohol drinking	Yes	91 (24.1%)	44 (35.2%)	0 (0%)	0.018^*^	83 (26.7%)	52 (26.4%)	0.942	135 (26.6%)
	No	286 (75.9%)	81 (64.8%)	6 (100%)		228 (73.3%)	145 (73.6%)		373 (73.4%)
Problematic internet use	Yes	317 (84.1%)	99 (79.2%)	5 (83.3%)	0.365^*^	252 (81%)	169 (85.8%)	0.165	421 (82.9%)
	No	60 (15.9%)	26 (20.8%)	1 (16.7%)		59 (19%)	28 (14.2%)		87 (17.1%)
Regular check-up	Yes	356 (94.4%)	112 (89.6%)	3 (50%)	0.002^*^	295 (94.9%)	176 (89.3%)	**0.020**	471 (92.7%)
	No	21 (5.6%)	13 (10.4%)	3 (50%)		16 (5.1%)	21 (10.7%)		37 (7.3%)

Chi-squared test (χ^2^) and Fisher's-exact test (*) had been used with a significance level (p-value) ≤ 0.05. The significant values are in bold font.

### HU-DBI Responses by Academic Year

The item no. 1 about dental anxiety (not worrying about visiting the dentist) had 24.2% disagreement with the second-year students having the highest level of anxiety 32.5%. The item no. 2 of brushing-induced gingival bleeding had only 4.1% of agreement; the first-year students had the highest agreement level (7.1%) and the final-year students had the lowest agreement level (1.2%) with a statistically significant difference (*p* = 0.054). The item no. 4 of noticing dental plaque had a low agreement level of 3.5% with no significant difference among the academic years. The item no. 5 of using child-sized toothbrush had a low agreement level 3.7% with the second-year students having the highest level (7%) while the first-year and final-year students had the lowest agreement level (1.2%).

The item no. 6 of self-efficacy that implies that one's oral health is predictable and controlled by hygiene behaviors had a high disagreement level of 94.1%; the first-year students had the highest agreement level (11.8%) and the final-year students had the lowest agreement level (3.5%) with a statistically significant difference (*p* = 0.044). The item no. 7 of being bothered by the gingival color had the lowest agreement level of 1.8% with no significant difference among the academic years. Item no. 9 of careful brushing had the highest agreement level of 97.2%, with no significant difference among the academic years. Item no. 10 of receiving training for oral hygiene by a professional had an agreement level of 25% with no significant difference among the academic years.

Item no. 13 of worrying about halitosis had 44.7% of agreement; the first-year students had an agreement level of 51.8%, while the final-year students had 43.5% of agreement. Item no. 16 of using a disclosing agent to visualize dental plaque had 46.5% of agreement, with a statistically significant (*p* < 0.001) difference between the first-year (31.8%) and the final-year (60%) students. Item no. 18 of involving hard strokes while brushing had 13.8% of agreement, with a statistically significant (*p* < 0.001) difference between the first-year (31.8%) and the final-year (5.9%) students (Table 4).

**Table 4:** Responses of German dental students to HU-DBI original items, winter 2021, (*n* = 508).

**Variable**	**Outcome**	**First year** **(*n* = 85)**	**Second year** **(*n* = 114)**	**Third year** **(*n* = 112)**	**Fourth year** **(*n* = 112)**	**Fifth year** **(*n* = 85)**	**Total** **(*n* = 508)**	***p-value***
Item no. 1	Agree	65 (76.5%)	77 (67.5%)	86 (76.8%)	95 (84.8%)	62 (72.9%)	385 (75.8%)	**0.047**
Item no. 2	Disagree	79 (92.9%)	109 (95.6%)	108 (96.4%)	107 (95.5%)	84 (98.8%)	487 (95.9%)	0.421^*^
Item no. 3	Agree	39 (45.9%)	55 (48.2%)	39 (34.8%)	35 (31.3%)	28 (32.9%)	196 (38.6%)	0.029
Item no. 4	Agree	3 (3.5%)	3 (2.6%)	4 (3.6%)	4 (3.6%)	4 (4.7%)	18 (3.5%)	0.936^*^
Item no. 5	Agree	1 (1.2%)	8 (7%)	3 (2.7%)	6 (5.4%)	1 (1.2%)	19 (3.7%)	0.125^*^
Item no. 6	Disagree	75 (88.2%)	106 (93%)	108 (96.4%)	107 (95.5%)	82 (96.5%)	478 (94.1%)	0.096
Item no. 7	Agree	2 (2.4%)	1 (0.9%)	3 (2.7%)	2 (1.8%)	1 (1.2%)	9 (1.8%)	0.840^*^
Item no. 8	Disagree	69 (81.2%)	100 (87.7%)	93 (83%)	102 (91.1%)	72 (84.7%)	436 (85.8%)	0.269
Item no. 9	Agree	80 (94.1%)	113 (99.1%)	109 (97.3%)	110 (98.2%)	82 (96.5%)	494 (97.2%)	0.276^*^
Item no. 10	Disagree	72 (84.7%)	101 (88.6%)	94 (83.9%)	95 (84.8%)	70 (82.4%)	432 (85%)	0.784
Item no. 11	Agree	2 (2.4%)	8 (7%)	6 (5.4%)	6 (5.4%)	5 (5.9%)	27 (5.3%)	0.700^*^
Item no. 12	Agree	70 (82.4%)	93 (81.6%)	87 (77.7%)	92 (82.1%)	64 (75.3%)	406 (79.9%)	0.674
Item no. 13	Agree	44 (51.8%)	62 (54.4%)	48 (42.9%)	36 (32.1%)	37 (43.5%)	227 (44.7%)	**0.010**
Item no. 14	Disagree	48 (56.5%)	64 (56.1%)	62 (55.4%)	62 (55.4%)	45 (52.9%)	281 (55.3%)	0.992
Item no. 15	Disagree	81 (95.3%)	108 (94.7%)	106 (94.6%)	104 (92.9%)	83 (97.6%)	482 (94.9%)	0.696^*^
Item no. 16	Agree	27 (31.8%)	40 (35.1%)	48 (42.9%)	70 (62.5%)	51 (60%)	236 (46.5%)	**<0.001**
Item no. 17	Agree	17 (20%)	17 (14.9%)	15 (13.4%)	14 (12.5%)	12 (14.1%)	75 (14.8%)	0.644
Item no. 18	Agree	27 (31.8%)	14 (12.3%)	15 (13.4%)	9 (8%)	5 (5.9%)	70 (13.8%)	**<0.001**
Item no. 19	Agree	25 (29.4%)	17 (14.9%)	24 (21.4%)	27 (24.1%)	25 (29.4%)	118 (23.2%)	0.081
Item no. 20	Agree	72 (84.7%)	103 (90.4%)	94 (83.9%)	95 (84.8%)	74 (87.1%)	438 (86.2%)	0.639

Chi-squared test (χ^2^) and Fisher's exact test (*) had been used with a significance level (p-value) ≤ 0.05. The significant values are in bold font.

### HU-DBI Responses by Gender and Clinical Experience

Clinical students (79.7%) and males (80.8%) had higher agreement levels for item no. 1 of dental anxiety (not worrying about visiting the dentist) compared with preclinical students (73.3%) and females (74%) without a statistical significance. Item no. 3 of worrying about teeth color had significantly (*p* = 0.015 and 0.005) higher agreement levels among preclinical students (42.8%) and females (42.2%) than clinical students (32%) and males (28%), respectively. Item no. 8 of the perceived decline of oral hygiene had higher levels of disagreement among clinical students (88.3%) and males (88.8%) than preclinical students (84.2%) and females (84.9%) without a statistical significance, respectively.

Item no. 13 of worrying about halitosis had a significantly agreement level (*p* = 0.006) among preclinical students (49.5%) than their clinical peers (37.1%). Similarly, item no. 18 of involving hard strokes while brushing had a significantly agreement level (*p* < 0.001) among preclinical students (18%) than their clinical peers (7.1%). On the other side, item no. 16 of using a disclosing agent to visualize dental plaque had a significantly agreement level (*p* < 0.001) among clinical students (61.4%) than their preclinical peers (37%). No statistically significant difference between females and males was found for items no. 13, 16, or 18. Males (20%) had a significantly level of agreement (*p* = 0.030) for item no. 17 of using a toothbrush with hard bristles than females (12.2%) (Table 5).

**Table 5:** Responses of German dental students to HU-DBI original items, winter 2021, (*n* = 508).

**Variable**	**Outcome**	**Female** **(*n* = 377)**	**Male** **(*n* = 125)**	**Gender-diverse** **(*n* = 6)**	***p-value***	**Preclinical** **(*n* = 311)**	**Clinical** **(*n* = 197)**	***p-value***
Item no. 1	Agree	279 (74%)	101 (80.8%)	5 (83.3%)	0.292^*^	228 (73.3%)	157 (79.7%)	0.102
Item no. 2	Disagree	366 (97.1%)	118 (94.4%)	3 (50%)	<0.001^*^	296 (95.2%)	191 (97%)	0.327
Item no. 3	Agree	159 (42.2%)	35 (28%)	2 (33.3%)	0.013^*^	133 (42.8%)	63 (32%)	**0.015**
Item no. 4	Agree	13 (3.4%)	2 (1.6%)	3 (50%)	<0.001^*^	10 (3.2%)	8 (4.1%)	0.615
Item no. 5	Agree	15 (4%)	3 (2.4%)	1 (16.7%)	0.171^*^	12 (3.9%)	7 (3.6%)	0.860
Item no. 6	Disagree	357 (94.7%)	116 (92.8%)	5 (83.3%)	0.231^*^	289 (92.9%)	189 (95.9%)	0.160
Item no. 7	Agree	5 (1.3%)	3 (2.4%)	1 (16.7%)	0.053^*^	6 (1.9%)	3 (1.5%)	1.000^*^
Item no. 8	Disagree	320 (84.9%)	111 (88.8%)	5 (83.3%)	0.445^*^	262 (84.2%)	174 (88.3%)	0.199
Item no. 9	Agree	367 (97.3%)	122 (97.6%)	5 (83.3%)	0.199^*^	302 (97.1%)	192 (97.5%)	0.811
Item no. 10	Disagree	317 (84.1%)	110 (88%)	5 (83.3%)	0.466^*^	267 (85.9%)	165 (83.8%)	0.519
Item no. 11	Agree	20 (5.3%)	7 (5.6%)	0 (0%)	1.000^*^	16 (5.1%)	11 (5.6%)	0.830
Item no. 12	Agree	299 (79.3%)	101 (80.8%)	6 (100%)	0.673^*^	250 (80.4%)	156 (79.2%)	0.743
Item no. 13	Agree	171 (45.4%)	53 (42.4%)	3 (50%)	0.790^*^	154 (49.5%)	73 (37.1%)	**0.006**
Item no. 14	Disagree	212 (56.2%)	67 (53.6%)	2 (33.3%)	0.504^*^	174 (55.9%)	107 (54.3%)	0.718
Item no. 15	Disagree	361 (95.8%)	117 (93.6%)	4 (66.7%)	0.023*	295 (94.9%)	187 (94.9%)	0.973
Item no. 16	Agree	177 (46.9%)	55 (44%)	4 (66.7%)	0.517^*^	115 (37%)	121 (61.4%)	**<0.001**
Item no. 17	Agree	46 (12.2%)	25 (20%)	4 (66.7%)	<0.001^*^	49 (15.8%)	26 (13.2%)	0.428
Item no. 18	Agree	49 (13%)	18 (14.4%)	3 (50%)	0.053^*^	56 (18%)	14 (7.1%)	**<0.001**
Item no. 19	Agree	93 (24.7%)	23 (18.4%)	2 (33.3%)	0.236^*^	66 (21.2%)	52 (26.4%)	0.178
Item no. 20	Agree	327 (86.7%)	107 (85.6%)	4 (66.7%)	0.283^*^	269 (86.5%)	169 (85.8%)	0.821

Chi-squared test (χ^2^) and Fisher's exact test (*) had been used with a significance level (p-value) ≤ 0.05. The significant values are in bold font.

### Determinants of HU-DBI Score

**Figure 1:**
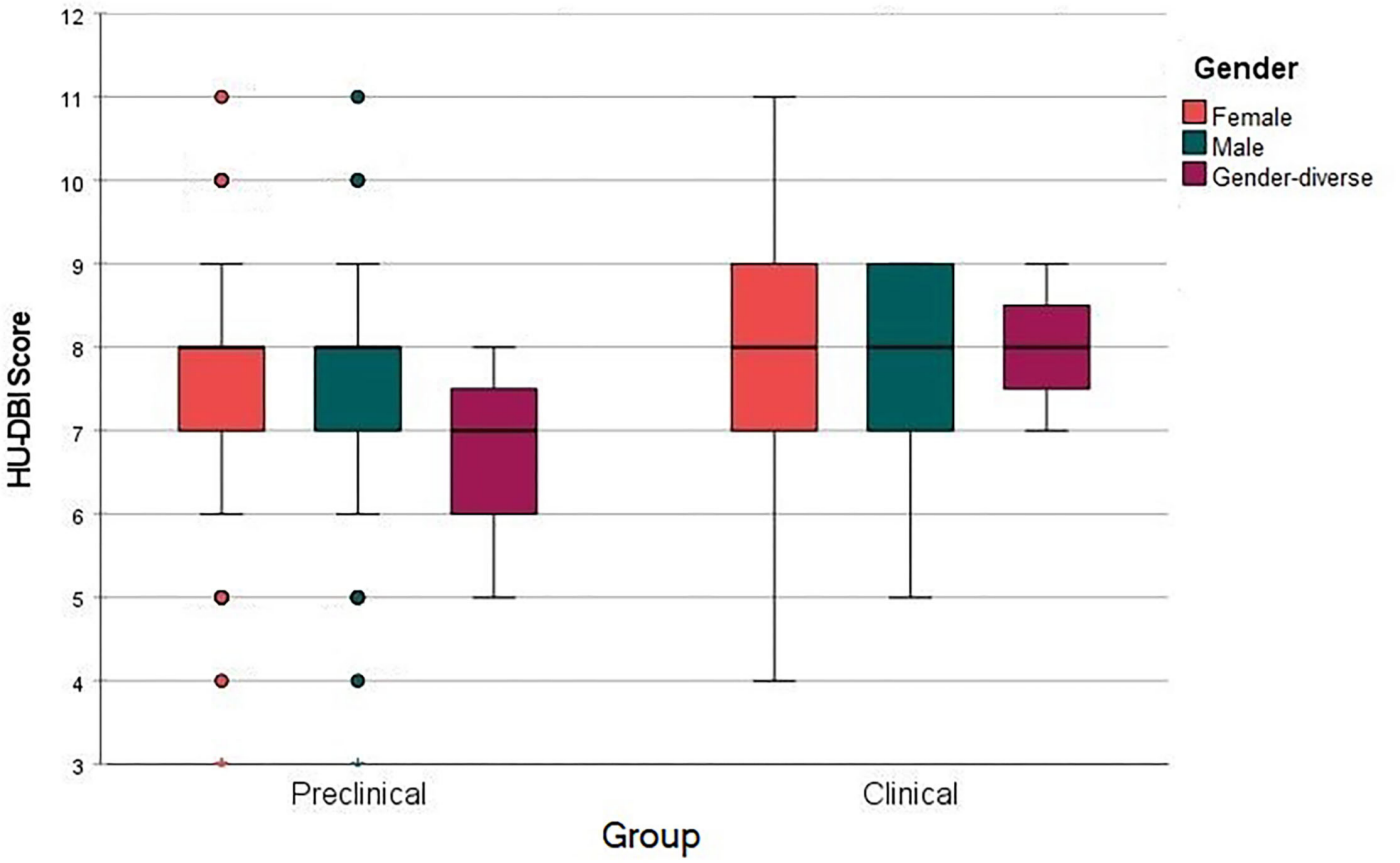
HU-DBI score of german dental students clustered by gender and clinical experience, winter 2021, (*n* = 508).

The overall HU-DBI score was 7.67 ± 1.32 (min. – max.: 3 – 11), which was composed of the three KAB elements: knowledge 3.85 ± 0.75 (1 – 5), attitudes 1.55 ± 0.61 (0 – 3), and behaviors 2.27 ± 0.71 (0 – 4). Females had the highest HU-DBI score (7.70 ± 1.33), followed by males (7.59 ± 1.29) and gender-diverse students (7.33 ± 1.37). Nevertheless, the gender-based differences were not statistically significant (*p* = 0.683) (Figure 1).

**Figure 2:**
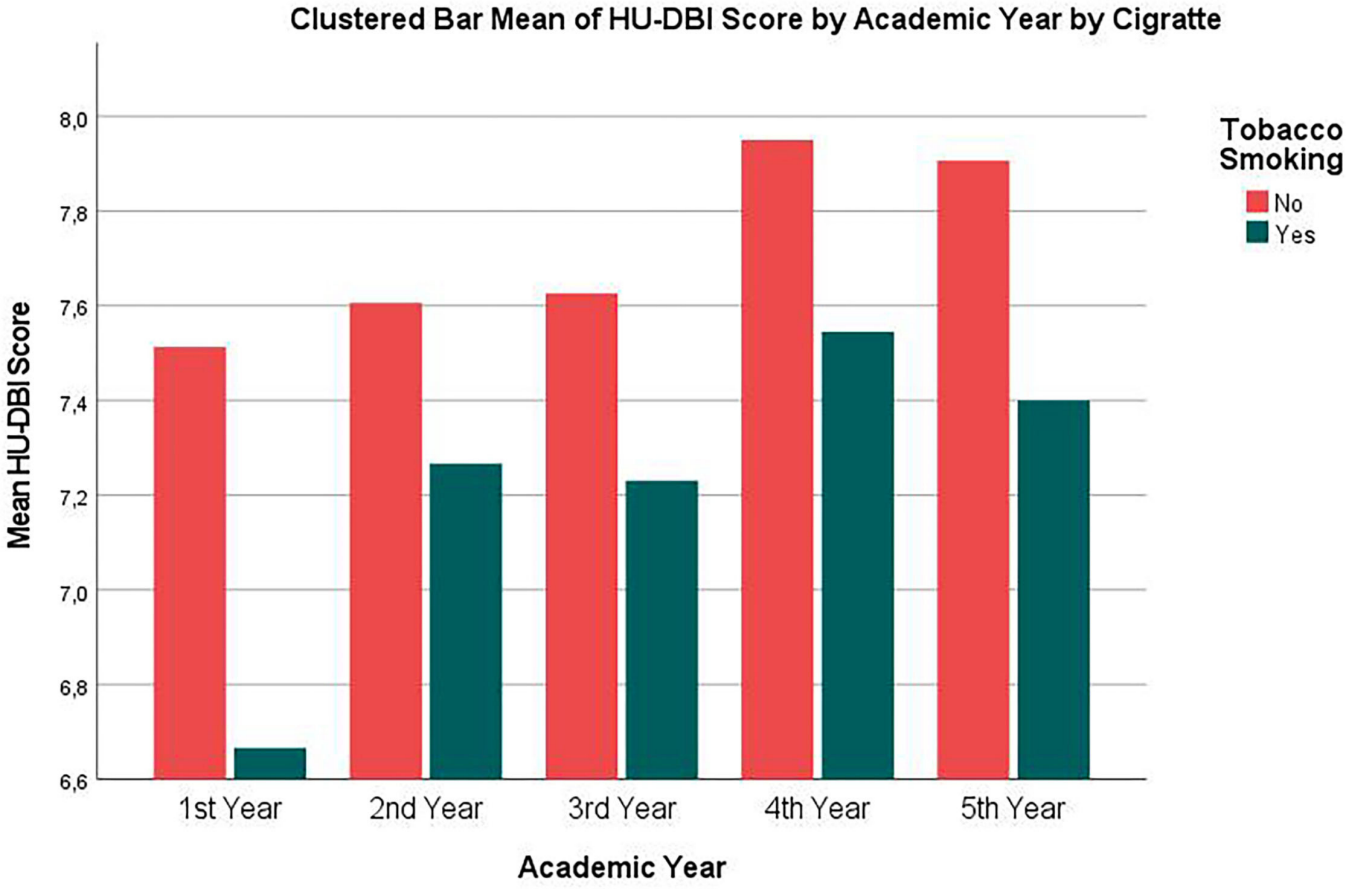
HU-DBI score of german dental students' clustered by tobacco smoking and academic year, winter 2021, (*n* = 508).

The behaviors-index score and the overall HU-DBI score had significantly risen through the five academic years. The final-year students had a significantly (*p* = 0.006) higher HU-DBI score (7.85 ± 1.31) compared with the first-year students (7.42 ± 1.43). Similarly, the clinical students had a significantly (*p* = 0.003) higher HU-DBI score (7.88 ± 1.26) than their preclinical peers (7.53 ± 1.34) (Figure 2).

Smokers had significantly (*p* = 0.041, 0.006, and 0.010) lower knowledge-index score (3.67 ± 0.71 *vs*. 3.87 ± 0.76), behaviors-index score (2.02 ± 0.73 *vs*. 2.30 ± 0.70), and overall HU-DBI score (7.24 ± 1.37 *vs*. 7.72 ± 1.31) than non-smokers, respectively (Figure 2).

The students who drink alcohol at least once a week and those who reported problematic internet use had lower overall HU-DBI scores than their counterparts; however, these differences were not statistically significant. The students who reported regular dental check-ups had a significantly (*p* < 0.001) higher knowledge-index score (3.88 ± 0.72 *vs*. 3.41 ± 0.99) and a nearly significant (*p* = 0.058) higher overall HU-DBI score (7.70 ± 1.31 *vs*. 7.27 ± 1.47), respectively (Table 6).

**Table 6:** Knowledge, attitudes, behaviors and total HU-DBI score of German dental students, winter 2021, (*n* = 508).

**Variable**	**Outcome**	**Knowledge** **(0–5)**	***p-value***	**Attitudes** **(0–3)**	***p-value***	**Behaviors** **(0–4)**	***p-value***	**HU-DBI** **(0–12)**	***p-value***
Gender	Female	3.86 ± 0.74	0.207	1.56 ± 0.61	0.287	2.27 ± 0.70	0.114	7.70 ± 1.33	0.683
	Male	3.83 ± 0.75		1.52 ± 0.61		2.24 ± 0.69		7.59 ± 1.29	
	Gender-diverse	3.17 ± 1.17		1.17 ± 0.75		3.00 ± 0.89		7.33 ± 1.37	
Academic year	1^st^ Year	3.84 ± 0.84	0.288	1.47 ± 0.65	0.648	2.12 ± 0.68	**<0.001**	7.42 ± 1.43	**0.006**
	2^nd^ Year	3.82 ± 0.67		1.56 ± 0.61		2.18 ± 0.65		7.56 ± 1.34	
	3^rd^ Year	3.79 ± 0.76		1.57 ± 0.63		2.21 ± 0.72		7.58 ± 1.29	
	4^th^ Year	3.88 ± 0.73		1.56 ± 0.57		2.46 ± 0.71		7.91 ± 1.22	
	5^th^ Year	3.93 ± 0.78		1.55 ± 0.63		2.36 ± 0.74		7.85 ± 1.31	
Clinical experience	Preclinical	3.81 ± 0.75	0.165	1.54 ± 0.63	0.948	2.18 ± 0.68	**<0.001**	7.53 ± 1.34	**0.003**
	Clinical	3.90 ± 0.75		1.56 ± 0.59		2.42 ± 0.72		7.88 ± 1.26	
Tobacco smoking	Yes	3.67 ± 0.71	**0.041**	1.55 ± 0.65	0.880	2.02 ± 0.73	**0.006**	7.24 ± 1.37	**0.010**
	No	3.87 ± 0.76		1.55 ± 0.61		2.30 ± 0.70		7.72 ± 1.31	
Alcohol drinking	Yes	3.86 ± 0.66	0.824	1.55 ± 0.63	0.958	2.24 ± 0.69	0.376	7.64 ± 1.24	0.509
	No	3.84 ± 0.79		1.55 ± 0.61		2.28 ± 0.71		7.68 ± 1.35	
Problematic internet use	Yes	3.83 ± 0.78	0.232	1.54 ± 0.61	0.715	2.26 ± 0.72	0.709	7.63 ± 1.34	0.339
	No	3.95 ± 0.59		1.57 ± 0.64		2.31 ± 0.65		7.84 ± 1.22	
Regular check-up	Yes	3.88 ± 0.72	**<0.001**	1.55 ± 0.61	0.883	2.27 ± 0.71	0.702	7.70 ± 1.31	0.058
	No	3.41 ± 0.99		1.51 ± 0.61		2.35 ± 0.72		7.27 ± 1.47	

*Mann-Whitney test (U), Kruskal-Wallis test (H), and Jonckheere-Terpstra test (JT) had been used with a significance level (p-value) ≤ 0.05. The significant values are in bold font*.

### Year-Over-Year Analysis

To evaluate the year-over-year changes of HU-DBI scores, the pairwise comparison through the Mann-Whitney test (*U*) was used; it revealed that the only significant change occurred between the third and the fourth year in terms of behaviors-index score (*p* = 0.006) and overall HU-DBI score (*p* = 0.034) (Table 7).

**Table 7:** Pairwise comparison of oral health knowledge, attitudes, behaviors and total HU-DBI score across consecutive academic years, winter 2021, (*n* = 508).

**Pair**	**Knowledge**		**Attitudes**		**Behaviors**		**HU-DBI**	
	**Mean rank**	***p-value***	**Mean rank**	***p-value***	**Mean rank**	***p-value***	**Mean rank**	***p-value***
1^st^ Year *vs*. 2^nd^ Year	102.01 / 98.50	0.624	95.47 / 103.38	0.280	97.42 / 101.92	0.541	95.57 / 103.30	0.335
2^nd^ Year *vs*. 3^rd^ Year	113.76 / 113.23	0.942	113.57 / 113.43	0.985	111.83 / 115.20	0.668	114.84 / 112.14	0.748
3^rd^ Year *vs*. 4^th^ Year	108.99 / 116.01	0.343	113.11 / 111.89	0.873	101.65 / 123.35	**0.006**	103.59 / 121.41	**0.034**
4^th^ Year *vs*. 5^th^ Year	97.55 / 100.91	0.639	99.76 / 97.99	0.807	102.38 / 94.55	0.294	100.23 / 97.38	0.719

*Mann-Whitney test (U) had been used with a significance level (p-value) ≤ 0.05. The significant values are in bold font*.

**Figure 3:**
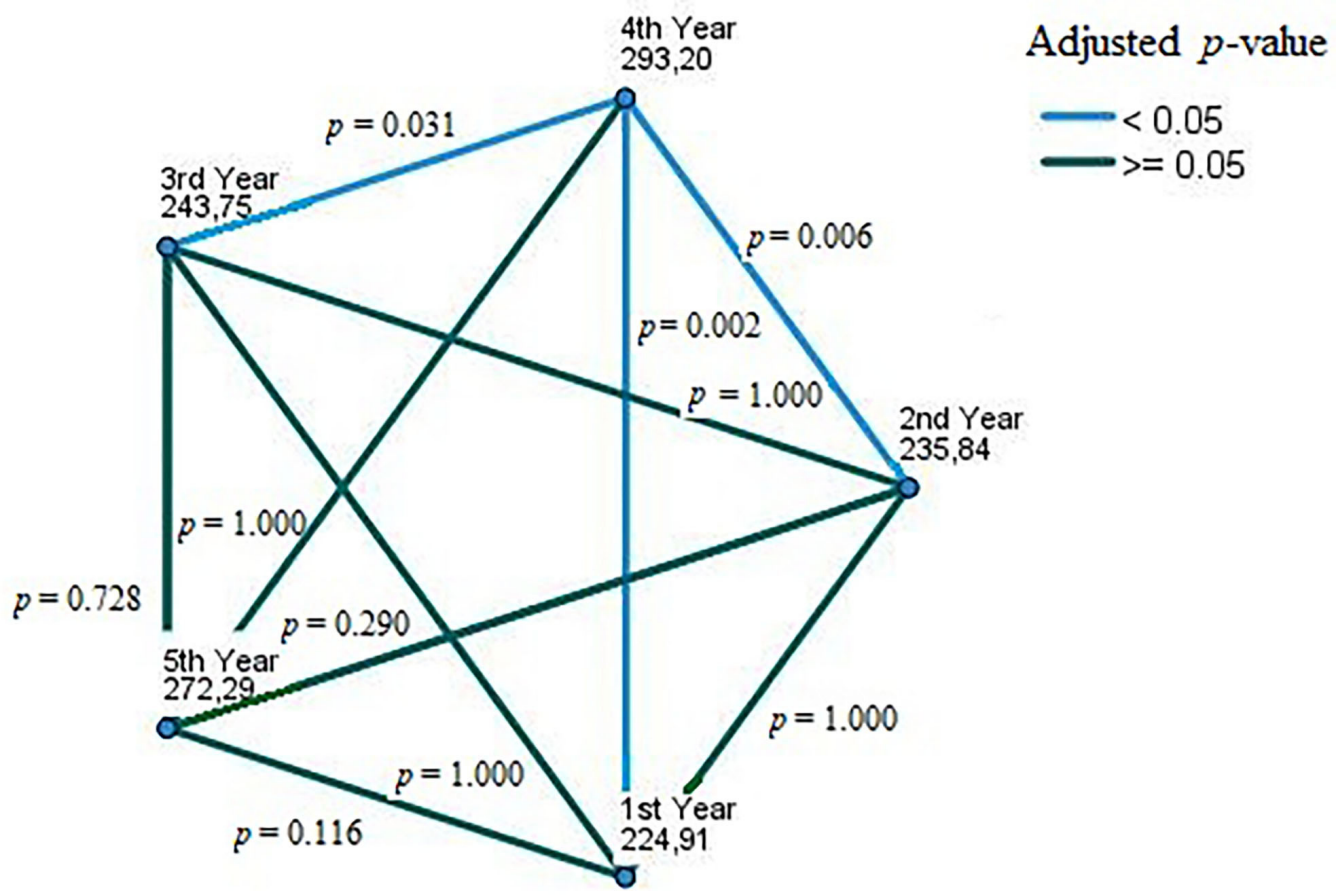
Pairwise comparison of german dental students' oral health behaviors across academic years, winter 2021, (*n* = 508). Each node shows the sample average rank of academic years. The adjusted *p*-value is calculated using Bonferroni error correction.

On performing a pairwise comparison of the behaviors-index score using Jonckheere-Terpstra test (*JT*), the differences between fourth-year *vs*. third-year (Adjusted Significance = 0.031), fourth-year *vs*. second-year (*Adj. p* = 0.006), and fourth-year vs. first-year (*Adj. p* = 0.002) were statistically significant (Figure 3).

### Determinants of Oral Hygiene Training, Smoking, and Regular Check-Up

Binary logistic regression was used to evaluate the predictors of receiving oral hygiene training by a professional (item no. 10). The regression analysis found that disagreement with items no. 2 (brushing-induced gingival bleeding), no. 6 (self-efficacy), no. 8 (perceived decline of oral health), and no. 18 (strong strokes), and agreement with items no. 12 (post-brushing checking) and no. 20 (positive feedback of the treating dentist) could significantly predict the oral hygiene training item (Table 8).

**Table 8:** Regression analysis of oral hygiene training predictors among German dental students, winter 2021, (*n* = 508).

**Predictor**	**B (SE)**	**Wald**	**OR (CI 95%)**	***p-value***
Item no. 2: disagree *vs*. agree	1.33 (0.47)	8.11	3.79 (1.51–9.49)	**0.004**
Item no. 6: disagree *vs*. agree	1.14 (0.41)	7.73	3.12 (1.40–6.96)	**0.005**
Item no. 8: disagree *vs*. agree	0.78 (0.31)	6.41	2.17 (1.19–3.96)	**0.011**
Item no. 12: agree *vs*. disagree	1.18 (0.27)	19.48	3.26 (1.93–5.51)	**<0.001**
Item no. 18: agree *vs*. disagree	0.62 (0.32)	3.88	1.87 (1.003–3.473)	**0.049**
Item no. 20: agree *vs*. disagree	0.62 (0.32)	3.88	1.87 (1.00–3.47)	**0.049**

*Binary logistic regression had been used with a significance level (p-value) ≤ 0.05. B, Unstandardized co-efficient Alexandria University; SE , standard error; Wald, Wald; OR , Odds Ratio; CI, Confidence Interval*.

Moreover, the regression analysis found that disagreement with item no. 6 (self-efficacy), and agreement with items no. 9 (careful brushing), no. 12 (post-brushing checking), and no. 16 (using disclosing agents) could significantly predict the tobacco smoking behavior, as they were associated with lower odds of smoking. The students who reported drinking alcohol once per week had an increased odds ratio of tobacco smoking behavior (Table 9).

**Table 9:** Regression analysis of smoking tobacco predictors among German dental students, winter 2021, (*n* = 508).

**Predictor**	**B (SE)**	**Wald**	**OR (CI 95%)**	***p-value***
Item no. 6: disagree *vs*. agree	−1.14 (0.44)	6.69	0.32 (0.14–0.76)	**0.010**
Item no. 9: agree *vs*. disagree	−1.18 (0.61)	3.77	0.31 (0.09–1.01)	**0.052**
Item no. 12: agree *vs*. disagree	−0.67 (0.31)	4.76	0.51 (0.28–0.93)	**0.029**
Item no. 16: agree *vs*. disagree	−0.56 (0.29)	3.70	0.57 (0.32–1.01)	**0.054**
Alcohol drinking: yes *vs*. no	1.34 (0.29)	21.96	3.82 (2.18–6.69)	**<0.001**

*Binary logistic regression had been used with a significance level (p-value) ≤ 0.05. B, Unstandardized co-efficient Alexandria University; SE , standard error; Wald, Wald; OR , Odds Ratio; CI, Confidence Interval*.

The regression analysis found that disagreement with items no. 2 (brushing-induced gingival bleeding), no. 10 (receiving oral hygiene training), and no. 15 (postponing dental visits) and agreement with items no. 4 (noticing dental plaque), no. 9 (careful brushing), and no. 20 (positive feedback of the treating dentist) could significantly predict the regular dental check-up behavior (Table 10).

**Table 10:** Regression analysis of regular dental check-up predictors among German dental students, winter 2021, (*n* = 508).

**Predictor**	**B (SE)**	**Wald**	**OR (CI 95%)**	***p-value***
Item no. 2: disagree *vs*. agree	1.18 (0.58)	4.04	3.24 (1.03–10.17)	**0.044**
Item no. 4: agree *vs*. disagree	1.71 (0.56)	9.37	5.51 (1.85–16.40)	**0.002**
Item no. 9: agree *vs*. disagree	1.72 (0.62)	7.74	5.59 (1.66–18.78)	**0.005**
Item no. 10: disagree *vs*. agree	0.82 (0.39)	4.36	2.27 (1.05–4.91)	**0.037**
Item no. 15: disagree *vs*. agree	2.75 (0.44)	38.44	15.67 (6.57–37.39)	**<0.001**
Item no. 20: agree *vs*. disagree	0.93 (0.40)	5.55	2.54 (1.17–5.50)	**0.018**

*Binary logistic regression had been used with a significance level (p-value) ≤ 0.05. B, Unstandardized co-efficient Alexandria University; SE , standard error; Wald, Wald; OR , Odds Ratio; CI, Confidence Interval*.

## Discussion

Our study found that the overall HU-DBI score of dental students in Germany was 7.67 ± 1.32, which is higher than the overall score of dental students in other European countries such as Croatia (6.62 ± 1.54) (60), Finland (7.15 ± 1.13) (61), Greece (6.86 ± 1.83) (62), Lithuania (6.35 ± 1.43) (63), Poland (7.23 ± 1.45) (64), Romania (6.96) (65), and the United Kingdom (7.33) (66). On the other hand, German students' score was lower than their counterparts in the Netherlands (8.0 ± 1.19) (67), Portugal (7.74 ± 1.40) (67), and Switzerland (8.02 ± 1.27) (52).

According to the latest report of the Federal Statistical Office of Germany (Wiesbaden, Hesse, Germany), there had been 10,229 female and 5,346 male dental students enrolled in German universities during the winter semester of 2021/2022 (51). Our sample reflected the female domination of dental education in Germany; however, the female-to-male ratio of our participants (3.016: 1) was larger than the actual ratio of the target population (1.913: 1). Moreover, the present study found that female dental students in Germany had a slightly better oral health KAB than their male peers. Mekhemar et al. 2021 used a modified HU-DBI among a sample of 171 dental students in Germany and found that females had significantly better oral health attitudes than their male peers (47). Several HU-DBI-based studies of European dental students supported this finding of female superiority, e.g. Croatia (HU-DBI score: female *vs*. male = 6.58 *vs*. 6.17) (60), Finland (7.32 *vs*. 6.83) (61), Greece (7.13 *vs*. 6.48) (68), Poland (7.36 *vs*. 6.95) (64), Portugal (7.86 *vs*. 7.68) (69), Romania (7.24 *vs*. 6.50) (65), and the United Kingdom (7.4 *vs*. 7.21) (66). Nevertheless, the results of Dutch dental students did not agree with this female superiority trend of HU-DBI score (8.07 *vs*. 8.06) (67).

In Germany, population-based studies revealed that adult males suffer from periodontal diseases more frequently than females; however, females had higher caries experience than males (70). A recent cross-sectional study found that female adults had higher odds of seeking dental care compared with males (71). Another cross-sectional study for German armed force members' oral health concluded that females had better oral health outcomes depicted by a higher oral hygiene index score (OHI), lesser deep probing depths, less decayed and more filled teeth surfaces (72). In our sample, female students had a significantly higher agreement level with item no. 3 of worrying about teeth color than their male counterparts, 42.2 *vs*. 28%, respectively. Tin-Oo et al. (73) studied the factors that affect patient's satisfaction with their dental aesthetics and found that dissatisfaction with teeth color was significantly higher among female patients than males in Malaysia (73). Similarly, Strajnić et al. (74) concluded that female patients in Serbia were less satisfied with their general dental aesthetics (74). Females' dissatisfaction with their teeth color is echoed by their higher demand for teeth whitening services as found in Croatia, New Zealand, and Saudi Arabia, even among dental professionals (75–77).

Around 1.2% of our participants identified themselves as gender-diverse, which is lower than the estimated share of gender-diverse population in Germany that ranged between 1.9 and 7.4% according to various demographic surveys (78–80). According to the Dalia Research report, the German gender-diverse population is concentrated in young age groups, as they represented almost 12% of the people aged 14 – 29 years and about 6% of the people aged 30 – 65 years (79). However, pan-European studies show that Germany has one of the largest gender-diverse communities in the European Union (EU) with significant progress in terms of openness about being gender-diverse and decline of intolerance and prejudice, there was still 23% of gender-diverse people felt discriminated against within their workplaces and around one-fifth of trans and intersex people had been physically or sexually attacked during the last 5 years in Germany (81). In our sample, gender-diverse students had a lower HU-DBI score (7.33 ± 1.37) than heterosexual students (7.67 ± 1.32). The knowledge-index score and attitudes-index score of gender-diverse students were lower than their heterosexual peers; however, the behaviors-index score of gender-diverse students (3 ± 0.89) was significantly better than heterosexual students (2.26 ± 0.7). Russell et al. (82) pointed out to the lack of dental literature on oral health disparities of sexual and gender minorities whose diverse needs should be addressed by competent and accessible healthcare (82). Qualitative studies found that gender-diverse individuals were more susceptible to perceive discrimination, thus affecting their oral health-related quality of life and their oral healthcare access (83–85). While a few cohort studies in developed countries incorporated data about sexual orientation and oral health outcomes, e.g., National Health and Nutrition Examination Survey (NHANES), that enabled population-level analysis for the oral health needs of sexual and gender minorities, there is a lack of evidence on the oral health of the dynamic gender-diverse population in Germany (86). Given the under-representation of gender-diverse students in our sample, the present findings need to be interpreted with caution and they underline the need for addressing the oral health needs of sexual and gender minorities in Germany, especially among youth, through future epidemiologic studies (87).

Progressing from first-year to final-year within dental schools should not be only associated with the attainment of the theoretical knowledge and the professional skills that are required for providing clinical services, but it should also reflect an improvement of students' health beliefs and attitudes since the students will be the primary source of oral health-related information and they can have a key role in modifying their patients' health behaviors (88–91). Items no. 1 dental anxiety, no.3 worrying about teeth color, no. 16 of using a disclosing agent, and no. 18 of using hard strokes while brushing exhibited significantly gradual improvement through the five academic years. Item no. 3 of worrying about teeth color had a significantly higher agreement level among preclinical students than clinical students. In line with this result, El Mourad et al. (92) found that the first-year dental students in Saudi Arabia were significantly less satisfied with their teeth color than the final-year students (92).

Item no. 13 of worrying about halitosis had a significantly higher agreement level among preclinical students. Ashwath et al. (93) found that Indian dental students had substantial knowledge about halitosis and a high prevalence of self-perceived halitosis (93). Male dental students had higher levels of self-treatment for halitosis, while females had higher levels of mouth rinse use (93). In Libya and Pakistan, female dental students had higher levels of self-perceived halitosis, while Iraqi female dental students had lower levels of self-perceived halitosis compared with their male peers (94–96). Given the fact that the previous studies concluded that self-perceived halitosis among dental students was significantly associated with poor oral hygiene practices, it can be proposed that our clinical students had been less bothered by halitosis because they had exhibited a significantly better behaviors-index score (93–97). Therefore, advancing with dental education and improved oral health behaviors can reduce self-perceived halitosis among dental students.

Our clinical students had a significantly higher agreement level for item no. 16 (using disclosing agents) than preclinical students. The pairwise comparison revealed that difference between first-year *vs*. second year (*p* = 0.625), second-year *vs*. third-year (*p* = 0.232), and fourth-year *vs*. final-year (*p* = 0.722) students were not statistically significant in terms of using disclosing agents; however, the difference between third-year *vs*. fourth-year (*p*= 0.003) students was significant. The substantial shift from third- to fourth-year can be attributed to the courses of hygiene and prophylaxis that are typically delivered in German universities within the 6^th^ semester (third-year) or 7^th^ semester (fourth-year) (98–101). One can also put forward that this shift may have been enhanced by the course of periodontology which is usually situated within the same time interval (60).

Badovinac et al. (60) found that preclinical dental students in Croatia (6.33 ± 1.52) had a lower HU-DBI score than their clinical colleagues (6.88 ± 1.5) (60). Similarly, preclinical students had lower HU-DBI scores than clinical students in Lithuania (5.96 *vs*. 6.81) (63), Romania (6.95 *vs*. 7.35) (65), Saudi Arabia (5.8 *vs*. 7) (102), and Turkey (6 *vs*. 7.47) (103). Also, the present study found that preclinical students in Germany had significantly lower behaviors-index score (2.18 *vs*. 2.42) and HU-DBI score (7.53 *vs*. 7.88) compared with clinical students. On the other hand, a few HU-DBI-based studies found no difference between preclinical and clinical students such as the studies that were conducted in Egypt (46), India (104), and Sudan (105).

The hypothesis proposing that dental courses can impact students' behaviors positively is further supported by the findings of the year-over-year (YOY) analysis, which revealed that the sole significant shifts of German students' behaviors-index score (*p* = 0.006) and HU-DBI score (*p* = 0.034) occurred between the third- and fourth-year. Additionally, aggressive toothbrushing as a harmful behavior and one of the most common causes for the gingival recession was predicted to decline when comparing first-year and final-year students (106–108). The decline of aggressive toothbrushing as reported by item no. 18 was statistically significant only between the first- and second-year (*p* < 0.001); thus, suggesting an opportunity for curricular intervention that aims to impact students' beliefs and behaviors to be implemented at an earlier stage of dental education, i.e., during preclinical semesters (34, 45, 109). The current findings support the earlier suggestions for maximal behavior benefit through implementing preventive dentistry skills and dental public health concepts within multiple courses distributed across the full length of dental education, which needs to be in addition to the dedicated courses of preventive dentistry and dental public health (34, 43).

The Association for Dental Education in Europe (ADEE) recommends that one of the core elements for dental curricula in European universities should be “education in dental public health, preventive and community dentistry”; nevertheless, the concept and practice of dental public health in Germany is not well established in contrast to Anglo-Saxon and Scandinavian countries (110–112). Hugger et al. (38) identified one of the main barriers for implementing dental public health education in undergraduate German curricula, which is the fact that dental curricula have not been changed since 1955, and they are still bound with 65-year-old dental licensing regulations (38). For postgraduate specialization in dental public health, German dentists need training in isolated environments that are not connected with other dental specialities in order to pass the exam of the Academy for Public Health (Düsseldorf, North Rhine-Westphalia, Germany) (37). Therefore, there is a lack of dedicated dental public health departments and academic staff in German universities, representing another challenge for implementing dental public health education at the undergraduate level (113).

The prevalence of smoking in our sample was 11.4% which is lower than the reported prevalence of smoking among dental students (21%), medical students (28%), physicians (17.6%), and registered nurses (28.8%) in Germany (114–116). While smoking prevalence was not significantly different between preclinical and clinical students, males (20%) had a significantly (*p* < 0.001) higher prevalence of smoking compared to females (8.8%). According to Robert Koch Institute (Berlin, Germany), adult males (32.6%) had a higher smoking prevalence than females (26.9%) in Germany; and the young adults and the individuals with low socioeconomic status were more likely to be smokers (117). In our sample, the students who smoke at least once a week had significantly lower knowledge-index score (*p* = 0.041), behaviors-index score (*p* = 0.006), and overall HU-DBI score (*p* = 0.010) than their non-smoking colleagues. Smoking is associated with a lower health-related quality of life (HRQoL) and a higher demand for professional oral healthcare (118, 119). On the other hand, Almarek et al. (120) found that smoking students had a significantly higher HU-DBI score than non-smokers in Saudi Arabia (120).

The regression analysis revealed that agreement with item no. 9 (careful toothbrushing), no. 12 (post-brushing checking), and no. 16 (disclosing agent) had lower odds of tobacco smoking; thus, suggesting that there could be an association between oral health behaviors and smoking. Irregular dental care and dental anxiety were significantly associated with smoking among Swedish adults (121). In South Korea, the toothbrushing frequency of smoking adults was significantly lower than the frequency of non-smokers (122). Infrequent toothbrushing and tobacco smoking were significantly associated among Finnish adolescents (123). Yazdani et al. (124) found the same relationship among Iranian adolescents; therefore, they recommended that smoking cessation should be addressed by school-based oral health promotion programs (124).

Dentists can have an effective role in the fight against tobacco smoking through primary prevention, e.g., behavioral counseling, and primordial prevention, e.g., political support and advocacy for anti-smoking legal reformations (125). A pan-European study found that 67% of European dental schools had anti-smoking education implemented within their curricula; however, out of them, only 40% had practical skills training for smoking cessation (126). While German dental students were found to have an acceptable level of smoking-related knowledge, they exhibited insufficient self-perceived smoking cessation skills (114). Therefore, a recent controlled trial was designed to assess the effectiveness of educational interventions on German dental students' smoking cessation knowledge and skills (12). The trial of Vollath et al. (12) found that educational intervention was highly effective and managed to boost German students' smoking cessation knowledge and counseling skills; thus, calling for the inclusion of the newly developed course within the undergraduate curricula and suggesting that future research should evaluate the impact of these educational interventions on patient satisfaction in clinical settings (12).

Karacic et al. (127) found a strong correlation between German adolescents' health-related quality of life, mental health, and problematic internet use; thus, suggesting that problematic internet use negatively influences health outcomes and requires further research attention (127). A recent study for Saudi young adults concluded that poor oral hygiene behaviors were significantly associated with problematic internet use (128). The negative impact of problematic internet use on oral health KAB was not significant among our participants, which might be attributed to the fact that dental students represent an above-average subset of the general youth population in terms of oral health literacy.

### Strengths

To the best of the authors' knowledge, this study had the largest sample of dental students evaluated by HU-DBI in Germany. The impact of gender and clinical experience on dental students' KAB was feasibly assessed by the present study design. The year-over-year analysis through pairwise comparisons of academic years managed to track the gradual improvements of oral health behaviors among German dental students; thus, highlighting the role of undergraduate courses related to prevention and periodontology. The participation in this study was anonymous in order to control Hawthorne's effect and information bias. The potential association between oral health KAB and general health behaviors, e.g., smoking, drinking alcohol and problematic internet use were also evaluated.

### Limitations

The first limitation is attributed to the study's cross-sectional nature that hinders follow-up analysis for the changes of dental students' oral health KAB while they progress with their education. The second limitation is the gender imbalance of the recruited sample, as females were overrepresented while males and gender-diverse students were underrepresented. The third limitation is the lack of information about students' ethnic and cultural backgrounds that may impact their oral health KAB. The fourth limitation is the absence of comparison groups, e.g., non-dental or non-healthcare students. The fifth limitation is the lack of detailed information about the risk behaviors, e.g., smoking frequency, intensity, and duration.

### Implications

Given the results of the present study, preventive dentistry elements need to be integrated within German curricula at an earlier stage, and dental public health education should be effectively implemented with addressing gender-based oral health disparities. German dental curricula may also benefit from incorporating practical training on smoking cessation. The present study also suggests that future oral health research in Germany should focus on the oral health needs of the growing gender-diverse and immigrant populations.

## Conclusion

Overall, German dental students reported high levels of oral health KAB denoted by a mean HU-DBI score of 7.67 ± 1.32, which is higher than the vast majority of European students reported previously. Females had a slightly higher HU-DBI score, while gender-diverse students were under-represented in this study. Clinical students had a significantly higher HU-DBI score, especially in the domain of oral health behaviors, compared with preclinical students. A significant improvement in oral health behaviors and HU-DBI score was found between the third- *vs*. the fourth year, which corresponds to the period when prophylaxis, hygiene, and periodontology courses are delivered. Tobacco smoking was significantly associated with lower oral health knowledge, behaviors, and overall HU-DBI score. Problematic internet use and alcohol drinking had slightly lower HU-DBI scores. The findings of the present study call for early implementation of preventive dentistry elements in German curricula and addressing oral health needs of gender minorities in Germany by future epidemiologic studies.

## Data Availability Statement

The original contributions presented in the study are included in the article/supplementary materials, further inquiries can be directed to the corresponding author.

## Ethics Statement

The studies involving human participants were reviewed and approved by Ethics Committee of the Faculty of Medicine, Masaryk University under the reference number 48/2019. The patients/participants provided their written informed consent to participate in this study.

## Author Contributions

AR: conceptualization, software, formal analysis, writing—original draft preparation, and project administration. AR and MKr: methodology. SA and MB: validation. SA, MB, and H-PH: investigation. MKl, MKr, and SA: writing—review and editing. MKr: supervision. SA and H-PH: funding acquisition. All authors have read and agreed to the published version of the manuscript.

## Funding

This study was supported by Masaryk University grants no. MUNI/IGA/1104/2021 and MUNI/A/1402/2021. The work of AR and MKl was supported by the Inter-Excellence grant number LTC20031—Toward an International Network for Evidence-based Research in Clinical Health Research in the Czech Republic.

## Conflict of Interest

The authors declare that the research was conducted in the absence of any commercial or financial relationships that could be construed as a potential conflict of interest.

## Publisher's Note

All claims expressed in this article are solely those of the authors and do not necessarily represent those of their affiliated organizations, or those of the publisher, the editors and the reviewers. Any product that may be evaluated in this article, or claim that may be made by its manufacturer, is not guaranteed or endorsed by the publisher.
